# Comprehensive Analysis of N6-Methyladenosine-Related lncRNA Signature for Predicting Prognosis and Immune Cell Infiltration in Patients with Colorectal Cancer

**DOI:** 10.1155/2021/8686307

**Published:** 2021-10-28

**Authors:** Zhan Zhao, Ya-bing Yang, Xin-yuan Li, Xu-Guang Li, Xiao-dong Chu, Zheng-bin Lin, Yi-ran Zhang, Yan-guan Guo, Hui Ding, Yun-long Pan, Liang Wang, Jing-hua Pan

**Affiliations:** ^1^Department of General Surgery, The First Affiliated Hospital of Jinan University, Guangzhou 510632, China; ^2^School of Basic Medicine, Jinan University, Guangzhou 510632, China; ^3^Department of Abdominal Surgery, The Second Affiliated Hospital of Guangzhou University of Chinese Medicine, Guangzhou 510632, China; ^4^Department of Oncology, The First Affiliated Hospital of Jinan University, Guangzhou 510632, China

## Abstract

**Background:**

Colorectal cancer (CRC) is the third most common tumor worldwide. Aberrant N6-methyladenosine (m6A) modification can influence the progress of the CRC. Additionally, long noncoding RNA (lncRNA) plays a critical role in CRC and has a close relationship with m6A modification. However, the prognostic potential of m6A-related lncRNAs in CRC patients still remains to be clarified.

**Methods:**

We use “limma” R package, “glmnet” R package, and “survival” R package to screen m6A-related-lncRNAs with prognostic potential. Then, we comprehensively analysed and integrated the related lncRNAs in different TNM stages from TCGA database using the LASSO Cox regression. Meanwhile, the relationship between functional enrichment of m6A-related lncRNAs and immune microenvironment in CRC was also investigated using the TCGA database. A prognostic model was constructed and validated to determine the association between m6A-related lncRNAs in different TNM stages and the prognosis of CRC.

**Result:**

We demonstrated that three related m6A lncRNAs in different TNM stages were associated with the prognosis of CRC patients. Patients from the TCGA database were classified into the low-risk and the high-risk groups based on the expression of these lncRNAs. The patients in the low-risk group had longer overall survival than the patients in the high-risk group (*P* < 0.001). We further constructed and validated a prognostic nomogram based on these genes with a C-index of 0.80. The receiver operating characteristic curves confirmed the predictive capacity of the model. Meanwhile, we also found that the low-risk group has the correlation with the dendritic cell (DC). Finally, we discovered the relationship between the m6A regulators and the three lncRNAs.

**Conclusion:**

The prognostic model based on three m6A-related lncRNAs exhibits superior predictive performance, providing a novel prognostic model for the clinical evaluation of CRC patients.

## 1. Introduction

Colorectal cancer (CRC) is the third most common malignancy worldwide and has become a leading cause of cancer-related death [1, 2]. The prognosis of CRC is poor in advanced stages [3]. The 5-year survival rate of CRC varies across studies, ranging from 91.0% to 80.0% in histological stages I and II to 61.7-23.2% in histological stages III and IV [4, 5]. 42% of patients develop local recurrence or distant metastasis in stages II and III [6]. However, effective indicator for CRC development and prognosis is limited in the current times. Therefore, finding the key tumor biomarkers to predict the progress of CRC is needed.

At present, aberrant DNA methylation, abnormal histone modifications, and altered expression levels of various noncoding RNAs and long noncoding RNA (lncRNA) play an important role in the progress of tumors [7]. Among them, N6-methyladenosine (m6A) is the most common RNA modification, and the dynamic regulation of m6A modifications has been shown to be significantly related to gene expression [8–11]. M6A modifications occur via the m6A methyltransferases called “writers,” removed by the demethylases called “erasers,” and recognized by m6A-binding proteins called “readers” [9, 12–14]. Aberrant M6A modification promoted the progression of tumorigenesis in CRC by regulating the level of SOX2 transcripts, which can predict the poor prognosis in CRC [15]. However, whether the m6A modification can be used for early diagnosis and prognostic evaluation of colorectal cancer, which may facilitate the clinical evaluation of colorectal cancer patients.

Previous studies have found that m6A modifications can influence the binding between lncRNAs and specific DNA loci, affecting the progression of the tumor [16]. lncRNAs are defined as transcripts of more than 200 nucleotides that are not translated into proteins, but lncRNAs play different roles in mammals, such as regulating chromatin dynamics, gene expression, growth, differentiation, and development [17, 18]. For example, lncRNA CCAT2 can facilitate metastatic progression, increase chromosomal instability, and promote CRC tumorigenesis by activating the WNT pathway [19, 20]. lncRNA also can upregulate m6A regulators (YTHDF3) through a yes-associated protein- (YAP-) dependent manner and promote the progress of CRC patients [21]. In CRC, m6A-induced lncRNA RP11 can trigger the liver metastasis of CRC via posttranslational upregulation of Zinc-finger E-box-binding homeobox 1 (Zeb1) [22]. However, whether m6A-modified lncRNA could serve as a predictor for CRC prognosis remains unknown.

In our study, we integrated the information of CRC samples from TCGA and GEO database to comprehensively demonstrate the prognostic value of m6A-related lncRNAs and the relationship between them and TILs in CRC. We revealed that three different m6A-related lncRNAs (ALMS1-IT1, LINC01138, and ZEB1-AS1) can serve, respectively, as an independent factor in CRC prognosis.

## 2. Method and Materials

### 2.1. Data Acquisition

The RNA sequencing and corresponding clinical data were downloaded from the TCGA database (https://portal.gdc.cancer; including 568 CRC samples and 44 normal tissue samples) and the GEO database (http://www.ncbi.nlm.nih.gov/geo/; GSE39582 including 523 CRC samples). The somatic mutation data and copy number variation (CNV) data was acquired from TCGA database. A total of 24 regulators were extracted from the above datasets for identifying different m6A modification patterns mediated by m6A regulators. These 24 m6A regulators included 8 writers (METTL3, METTL14, METTL16, RBM15, RBM15B, WTAP, VIRMA, ZC3H13), 2 erasers (ALKBH5, FTO), and 14 readers (YTHDC1, YTHDC2, YTHDF1, YTHDF2, YTHDF3, IGF2BP1, HNRNPA2B1, HNRNPC, FMR1, LRPPRC, IGFBP1, IGFBP2, IGFBP3, RBMX). Since the TCGA and the GEO databases are publicly available and this study strictly followed access policies for databases and publication guidelines, ethical approval from a local ethics committee was not required.

### 2.2. Screening and Identifying Signature Associated with CRC Prognosis

The mRNA sequencing data from the TCGA database was matched with m6A-related genes. And the expression levels were identified between CRC tissues and adjacent nontumorous tissues. A coexpression analysis was conducted to identify the correlation of m6A-related gene expression with lncRNAs through “limma” R package. We derived the regulatory hypotaxis between the two factors with the correlation coefficient, and the network plot was depicted to visualize the correlation. Then, the differentially expressed genes (DEGs) between stage I-II and stage III-IV CRC patients were identified by the “limma” R package with a false discovery rate of <0.05. Univariate Cox analysis of overall survival (OS) was performed using the “survival” R package to screen m6A-related-lncRNAs with prognostic potential. The overlapping prognostic DEGs were incorporated into the LASSO Cox regression using the “glmnet” R package. According to the minimum criteria, penalty parameter (*λ*) was obtained by tenfold cross-validation. The risk score of each patient was calculated based on the expression of each gene and the corresponding regression coefficient: Risk score = SUM (expression of each gene × corresponding coefficient). CRC patients were divided into the low-risk and the high-risk groups according to the median of the risk score. Principal component analysis (PCA) was performed using the “prcomp” function of the “stats” R package based on signature gene expression in the TCGA database. In addition, t-SNE was performed by the “Rtsne” R package to investigate the distribution of the two groups.

### 2.3. Construction and Evaluation of the Predictive Nomogram

To determine whether the risk score was an independent prognostic predictor for OS compared to other clinical features in the TCGA database, univariate and multivariate Cox regression analyses were performed. The “rms” R package was used to construct a predictive nomogram and corresponding calibration maps based on independent predictive factors. Time-dependent receiver operating characteristic (ROC) curve analysis was performed to evaluate the predictive power of the nomogram using the “timeROC” R package. Patients from GSE39582 were analysed using the same formula as that for the TCGA database. ROC curves were generated to determine the sensitivity and specificity of the predictive nomogram.

### 2.4. Functional Enrichment Analysis

Gene Ontology (GO) analyses were performed. The *P* value was adjusted by the BH method. Single-sample gene set enrichment analysis (ssGSEA) in the “gsva” R package was used to assess the activity of 13 immune-related pathways and to calculate the infiltration scores of 16 immune cells.

### 2.5. Statistical Analysis

All statistical analyses were performed using the R software (version 4.0.3). The student's two-sided *t*-test was performed to compare gene expression between CRC tissues and adjacent nontumorous tissues. The OS of different groups was compared by Kaplan-Meier analysis followed by log-rank test. Mann–Whitney test was performed to compare the ssGSEA scores of immune pathways or cells between the two groups. All *P* values were two-tailed. A *P* value < 0.05 was considered statistically significant if not specified above.

## 3. Results

### 3.1. Landscape of Genetic Variation of m6A Regulators in Colorectal Cancer

**Figure 1:**
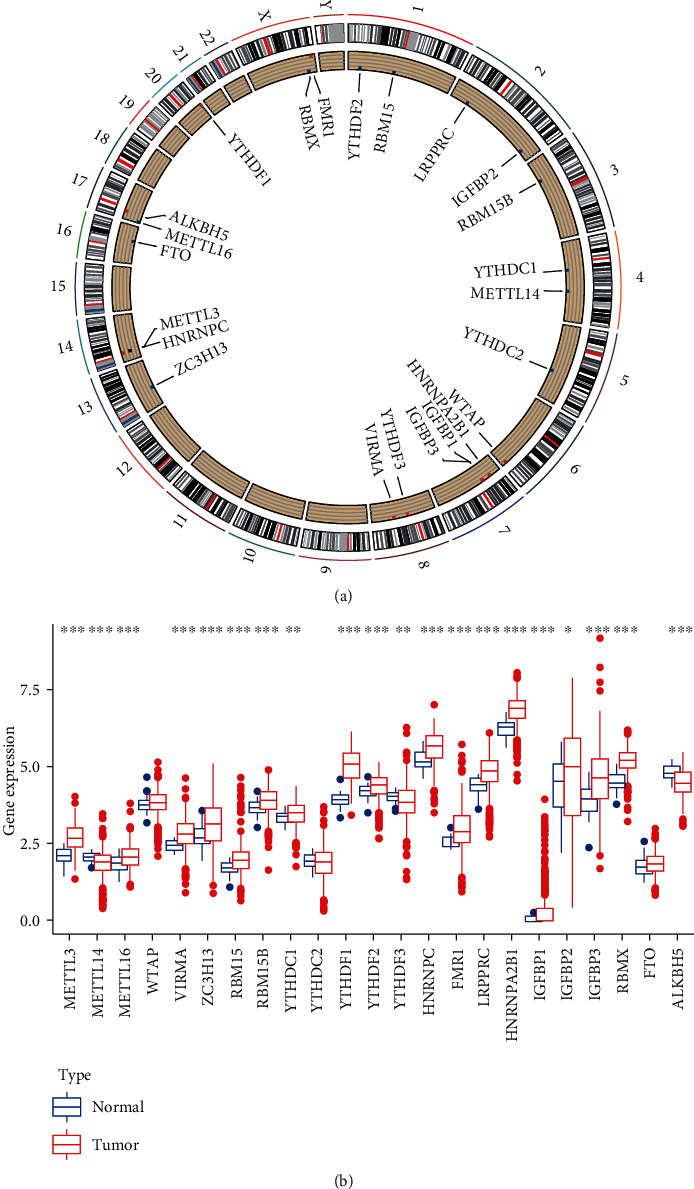
Landscape of genetic and expression variation of m6A regulators in colorectal cancer. (a) The location of copy number variation (CNV) alteration of m6A regulators on 24 chromosomes using TCGA datasets. (b) The expression of 24 m6A regulators between normal tissues and colorectal tissues. Tumor, red; normal, blue. The upper and lower ends of the boxes represented interquartile range of values. The lines in the boxes represented median value, and black dots showed outliers. The asterisks represented the statistical *P* value (^∗^*P* < 0.05; ^∗∗^*P* < 0.01; ^∗∗∗^*P* < 0.001).

A total of 24 m6A regulators including 8 writers, 2 erasers, and 14 readers were finally identified in this study. We first summarized the incidence of copy number variations and somatic mutations of 24 m6A regulators in CRC. The location of CNV alteration of m6A regulators on chromosomes is shown in Figure 1(a). Based on the expression of these 24 m6A regulators, we could completely distinguished CRC samples from normal samples in CRC tissues (Figure 1(b)). The above analyses presented the highly heterogeneity of genetic and expressional alteration landscape in m6A regulators between normal and CRC samples, indicating that the expression imbalance of m6A regulators played a crucial role in the CRC occurrence and progression.

### 3.2. Identification of m6A-Related lncRNAs in Prognosis Model

**Figure 2:**
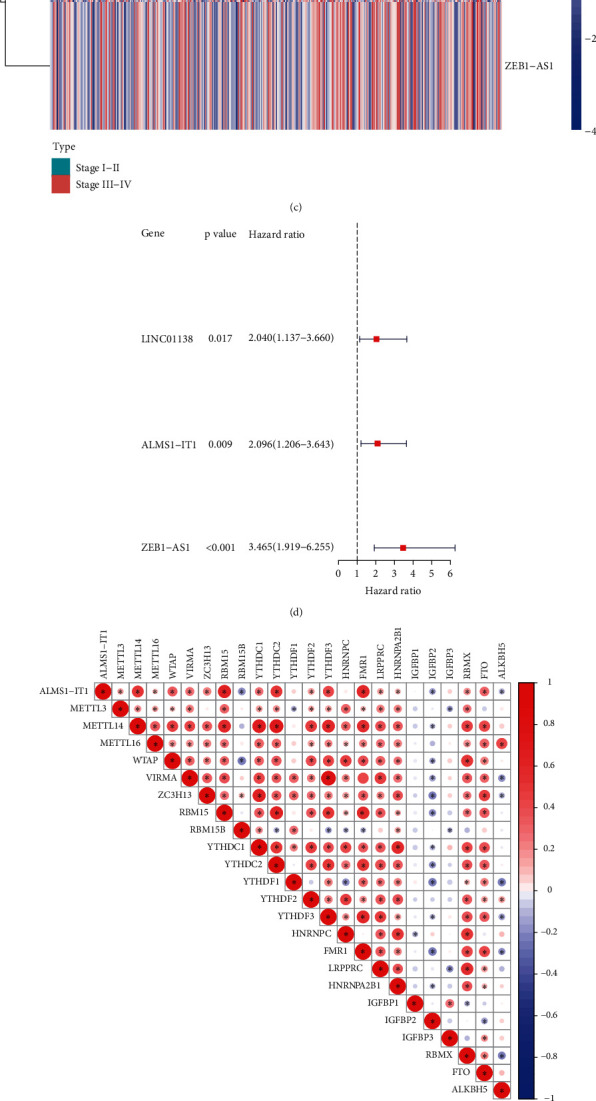
The relationship between prognostic m6A-related long noncoding RNAs (lncRNAs) and m6A regulators in colorectal cancers. (a) Network plot of correlation among m6A-related gene expression and lncRNAs. (b) Intersection analysis of m6A-related genes and prognostic model genes in CRC. (c) Heat map of the differences in the expression of m6A prognostic lncRNAs between stages I-II and stages III-IV. Red represents high expression, whereas blue represents low expression. The abscissa represents the sample, whereas the ordinate represents prognostic lncRNAs. (d) Forest plot of univariate Cox regression analysis. Data on prognostic lncRNAs were extracted, and the confidence intervals and hazard ratios were calculated. Red represents high-risk score. (e) Correlation analysis to analyse the correlation between target gene ALMS1-IT1 and prognostic m6A-lncRNAs in colorectal cancer. (f) Correlation analysis to analyse the correlation between target gene LINC01138 and prognostic m6A-related lncRNAs in colorectal cancer. (g) Correlation analysis to analyse the correlation between target gene ZEB1-AS1 and prognostic m6A-lncRNAs in colorectal cancer. Red means a positive correlation, whereas blue means a negative correlation; ^∗^ indicates a statistically significant difference.

To further understand the relationship among the m6A-related lncRNAs and prognosis models, the m6A-related gene expression data were extracted from the collated transcriptome data to distinguish between m6A and lncRNA. A network plot was drawn to identify the correlation among m6A-related gene expression and lncRNAs (Figure 2(a)). There were 53 out of 222 m6A-related lncRNAs differentially expressed in different stage. Among them, only 5 common lncRNAs were related with the prognosis. These 5 common lncRNAs were included in the LASSO Cox regression analysis. Finally, only 3 m6A-related lncRNAs (LINC01138, ALMS1-IT1, ZEB1-AS1) were included in our prognostic model. The differences between stages I-II and stages III-IV in the expression of the m6A-related prognostic lncRNAs were identified and are shown as heat map (Figures 2(b) and 2(c)). The results of univariate Cox regression analysis are shown in a forest plot that the three m6A-related lncRNAs were the important prognostic predictor for the TNM stage (Figure 2(d)). Finally, to clear the relationship between the potential m6A regulators and the screened lncRNAs, we analysed the correlation between the target gene and the m6A-related lncRNAs in CRC through correlation analysis. We found that the abovementioned target gene is related to m6A-related lncRNAs (*P* < 0.05). Expressions of ALMS1-IT1, LINC01138, and ZEB1-AS1 were associated with several m6A regulators, including RBM15, YDHTC2, FMR1, and FTO (Figures 2(e)–2(g)).

### 3.3. The Risk Scores Were Calculated Based on Three m6A-Related lncRNAs

**Figure 3:**
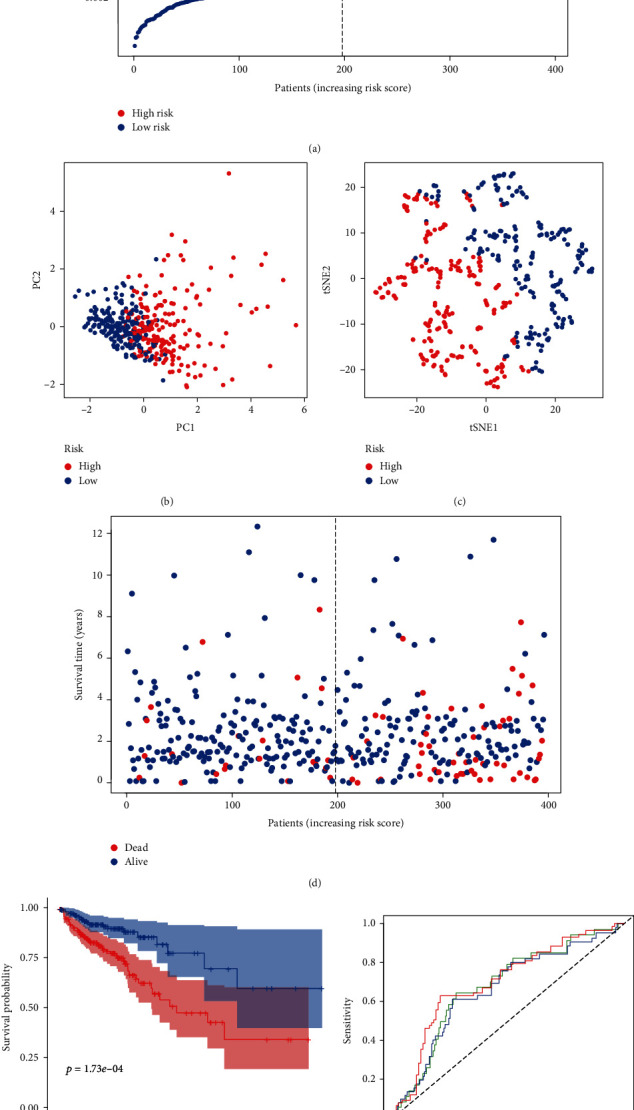
Prognostic analysis of the 3-gene risk level in the TCGA database. (a) The distribution and median value of the risk scores in the TCGA database. (b) PCA analysis of the TCGA database. (c) t-SNE analysis of the TCGA database. (d) The distributions of OS status, OS, and risk score in the TCGA database. (e) Kaplan-Meier curves for the OS of patients in the high-risk group and low-risk group in the TCGA database. (f) ROC curves of m6A-related lncRNAs for predicting 1/2/3 -year survival in the TCGA dataset.

To verify predictive value of our screened lncRNAs in CRC prognosis, we used the LASSO Cox regression to establish the risk scoring system analysing expression profile about three m6A-related lncRNAs. The risk score was calculated as follows: Risk score = SUM (0.000328 × LINC01138 + 0.00164 × ALMS1 − IT1 + 0.00279 × ZEB1 − AS1). Patients were classified into the low-risk and the high-risk groups according to the median cut-off value (Figure 3(a)). High-risk patients had a higher probability of death compared to the low-risk group (Figure 3(d)). T-SNE and PCA analysis showed that patients in different groups were distributed in two directions (Figures 3(b) and 3(c)). The Kaplan-Meier curve revealed that the prognosis of low-risk patients was significantly better than that of the high-risk group (Figure 3(e), *P* < 0.001), suggesting great sensitivity and specificity of the prognostic signature in predicting OS. A significant association between the three lncRNA genes and the prognosis of CRC patients was also observed. The ROC analysis also indicated that m6A-related lncRNA genes had a strong prognostic value for CRC patients in the TCGA dataset (1-year AUC = 0.679, 2-year AUC = 0.663, 3-year AUC = 0.699; Figure 3(f)).

**Figure 4:**
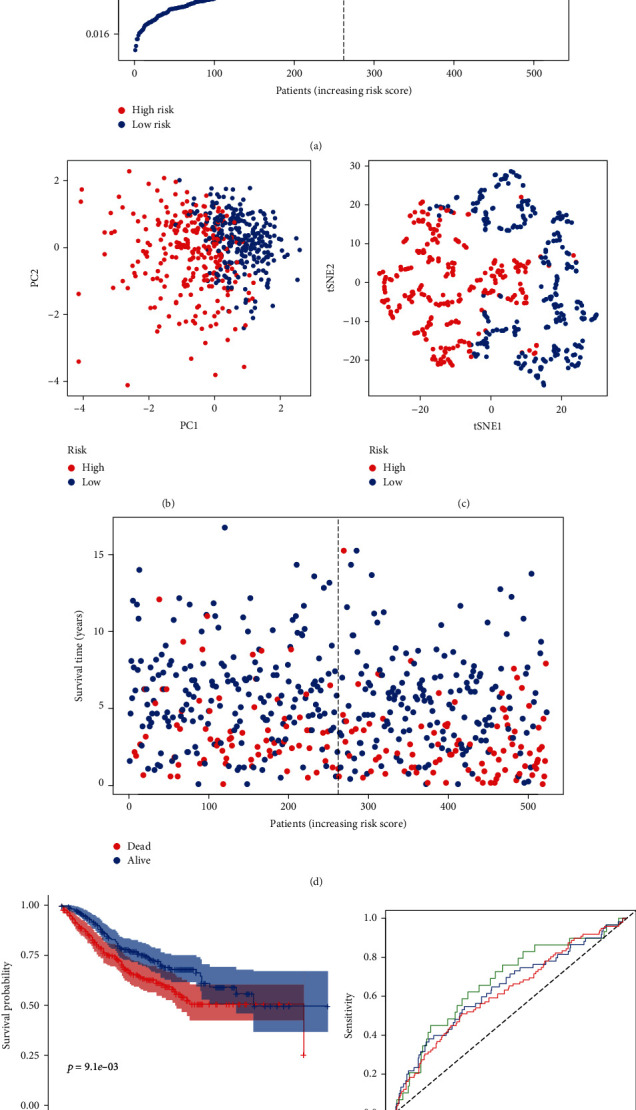
Prognostic analysis of the 3-gene risk level in the GEO database. (a) The distribution and median value of the risk scores in the GEO database. (b) PCA analysis of the GEO database. (c) t-SNE analysis of the GEO database. (d) The distributions of OS status, OS, and risk score in the GEO database. (e) Kaplan-Meier curves for the OS of patients in the high-risk group and low-risk group in the GEO database. (f) ROC curves of m6A-related lncRNAs for predicting 1/2/3-year survival in the GEO dataset.

These results showed that the m6A-related lncRNA genes had a robust and stable OS-predictive ability. Meanwhile, another group patients from GSE39582 were analysed using the same formula as that for the TCGA database. The result is as similar as the patients from TCGA (Figure 4).

### 3.4. Prognostic Risk Score Displayed Strong Correlations with Clinicopathological Features and Survival in CRC Patients

**Figure 5:**
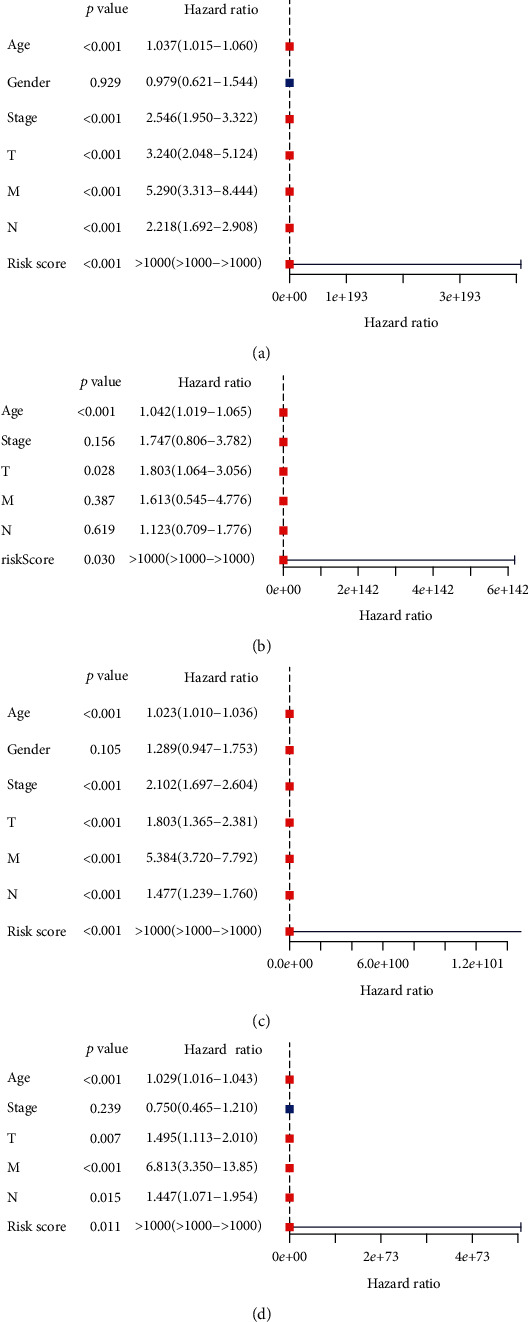
Cox regression analyses. (a, b) Results of the univariate and multivariate Cox regression analyses in the TCGA database. (c, d) Results of the univariate and multivariate Cox regression analyses in the GEO database.

To evaluate whether our model was independent of other clinical prognostic factors that could affect the patients' outcome, we tested the model with two independent sets of samples from different databases. In TCGA database, based on the univariate and multivariate Cox proportional hazards regression analyses, there were three independent predictors (age, T stage, and risk score) identified in the CRC. Univariate Cox proportional hazards regression analysis demonstrated that age (*P* < 0.001), TMN stage (*P* < 0.001), T stage (*P* < 0.001), N stage (*P* < 0.001), M stage (*P* < 0.001), and risk score (*P* < 0.001) had an impact upon OS (Figure 5(a)). Multivariate Cox proportional hazards regression analysis showed a significant correlation between age (HR = 1.042, *P* < 0.001), T stage (HR = 1.803, *P* = 0.028), and risk score (HR > 1000, P = 0.030) and OS in CRC patients (Figure 5(b)). In the GEO database, there were only two factors that were not the independent predictors (gender and TNM stage) in CRC. Univariate Cox proportional hazards regression analysis demonstrated that age (*P* < 0.001), TMN stage (*P* < 0.001), T stage (*P* < 0.001), N stage (*P* < 0.001), M stage (*P* < 0.001), and risk score (*P* < 0.001) had an impact upon OS (Figure 5(c)). Multivariate Cox proportional hazards regression analysis showed a significant correlation between age (HR = 1.029, *P* < 0.001), T stage (HR = 1.495, *P* = 0.007), N stage (HR = 1.447, *P* = 0.015), M stage (HR = 6.813, *P* < 0.001), and risk score (HR > 1000, *P* = 0.011) and OS in CRC patients (Figure 5(d)).

### 3.5. Construction and Validation of the Predictive Nomogram

**Figure 6:**
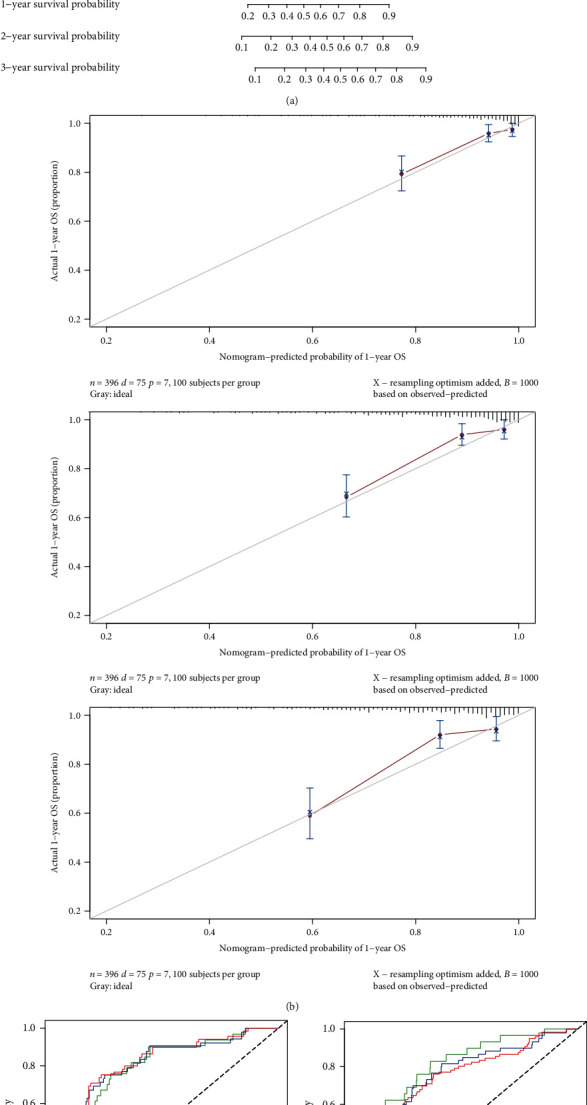
Construction and validation of a predictive nomogram. (a) The nomogram for predicting the OS of patients with CRC at 1, 2, and 3 years. (b) Calibration curves of the nomogram for OS prediction at 1, 2, and 3 years. (c) ROC curve analysis based on TCGA database. (d) ROC curve analysis based on GEO database.

Meanwhile, based on these independent prognostic factors, a nomogram was developed to quantify the prediction of individual survival probability for 1, 2, and 3 years (Figure 6(a)). The C-index of the nomogram was 0.80 (95% CI: 0.74–0.85). The calibration curves indicated great consistency between predicted OS and actual observation at 1, 2, and 3 years (Figure 6(b)).

Then, ROC curves were generated to verify the predictive value of the nomogram. The AUCs for 1-, 2-, and 3-year OS were 0.804, 0.807, and 0.805, respectively, in the TCGA database (Figure 6(c)). To examine the robustness of the model, we incorporated patients from the GEO database into the predictive model. The results showed that the AUCs for 1-, 2-, and 3-year OS with the nomogram were 0.798, 0.757, and 0.729, respectively (Figure 6(d)).

### 3.6. Functional Analysis of DEG

**Figure 7:**
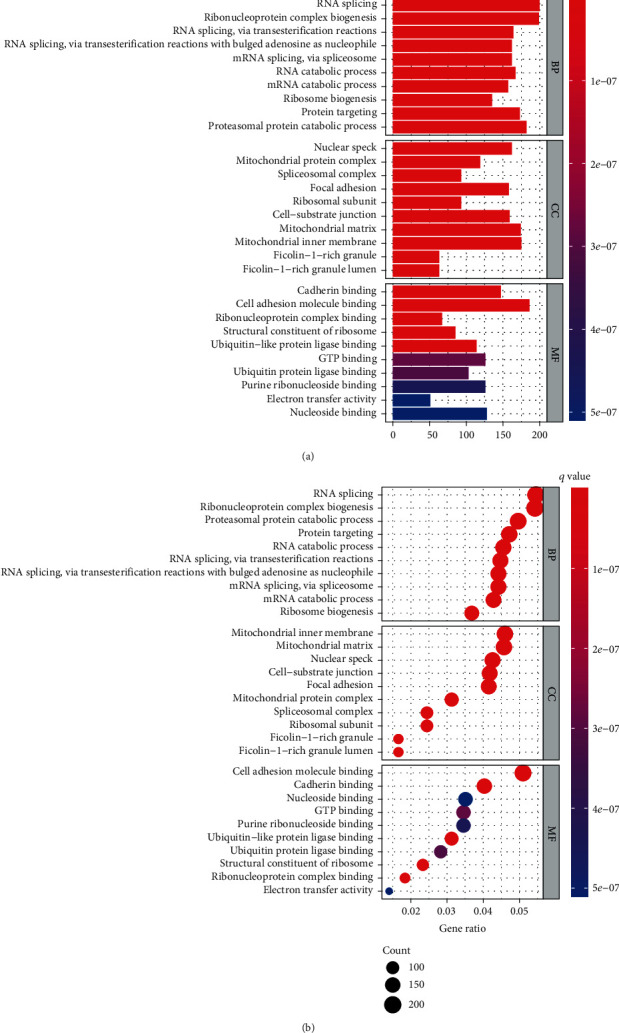
Representative results of functional analysis: (a) the most significant GO enrichment; (b) the most significant KEGG pathways.

To clarify the biological functions associated with the risk scores, we performed GO enrichment and KEGG pathway analyses using the DEGs. GO analysis demonstrated that DEGs were enriched in cell adhesion molecule binding and cadherin binding (Figure 7(a)). KEGG pathway analysis also revealed that cell adhesion molecule binding and cadherin binding were enriched in the TCGA database (Figure 7(b)).

### 3.7. Immune Infiltration and Immune Status of Patients in Different Risk Groups

**Figure 8:**
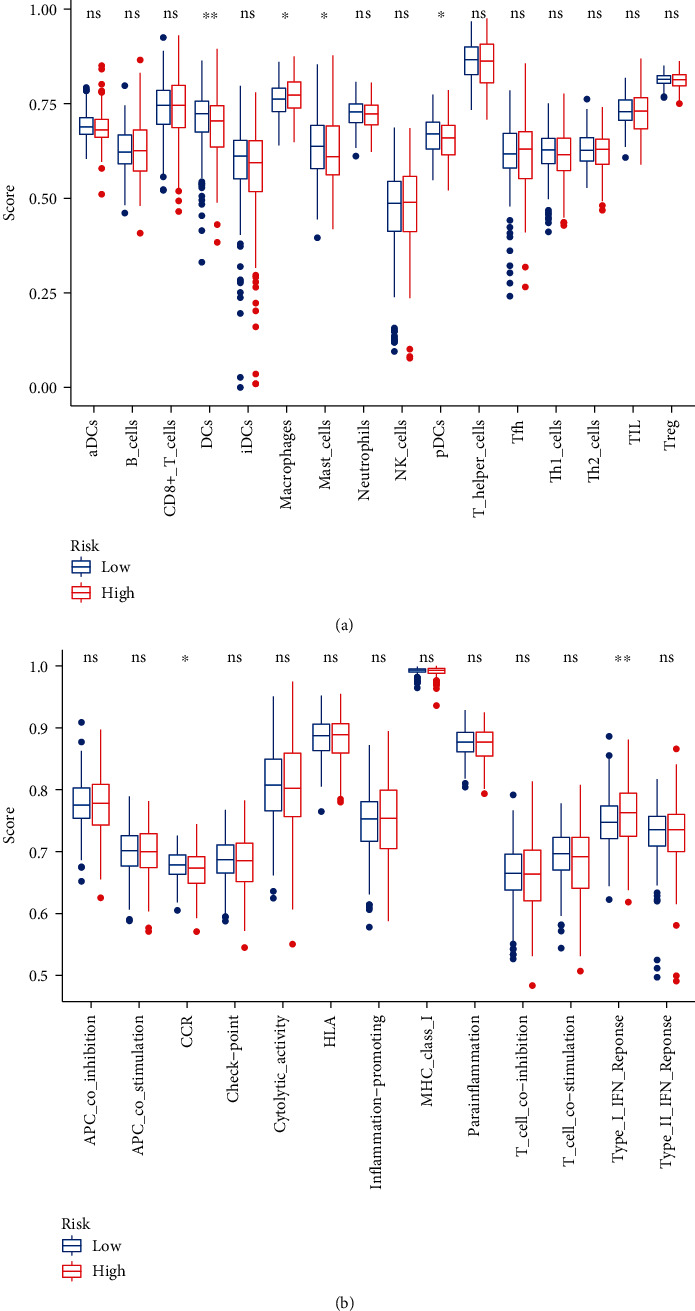
Results of immune infiltration in the TCGA database: (a) the scores of 16 immune cells; (b) the scores of 13 immune-related functions.

To investigate the correlation between risk score and immune status, the ssGSEA scores for different immune cell subsets, cell functions, and related pathways were quantified. The results showed that the scores of DC, plasmacytoid dendritic cells (pDC), and mast cells in high-risk patients were lower than those of the low-risk group (Figure 8(a)). Also, there were significant differences in type I interferon response and CCR in the TCGA database between the two groups (Figure 8(b)).

## 4. Discussion

In this study, we analysed the expression of 24 m6A-related lncRNA genes in CRC tissues and investigated their association with the OS of CRC patients using public databases. Three differentially expressed m6A-related lncRNA genes (ALMS1-IT1, LINC01138, and ZEB1-AS1) in different TNM stage CRC were associated with the prognosis. Patients with different expression levels of these genes showed different immune status and functional enrichment. Finally, we proposed a prognostic nomogram based on these genes, which exhibited great sensitivity and specificity in predicting OS. Our study showed that these three m6A-related lncRNAs can act as prognostic biomarkers in CRC. Meanwhile, it is the first prognostic model for different TNM stage CRC patients based on m6A-related lncRNA genes.

At present, highly expressed m6A modifications can increase the stability of the oncogenic lncRNA, which promotes cancer cell proliferation, invasion, and migration [23]. Meanwhile, lncRNA also can interact with the m6A regulators (METTL3) to suppress the stability of phosphatase and tensin homolog (PTEN) to facilitate gastric cancer (GC) progression [24]. Our study proposes a relationship between lncRNAs and m6A in the CRC and also found that the m6A-related lncRNAs had predicted effect on the progress and overall survival of the CRC, but the specific regulatory role among them remains further study.

Our results show that the abovementioned lncRNA genes highly express in the stage III-IV CRC. Previous studies found that ALMS1 was the lncRNA that targets the most mRNAs and proteins in head and neck squamous cell carcinoma (HNSCC), which related to the progression and prognosis of cancers [25]. Additionally, overexpression of ALMS1-IT1 promotes cell viability and heightened the number of colonies in lung cancer cells, promoting the malignant progression of lung adenocarcinoma [26]. LINC01138 regulates the expression of downstream genes through modulating protein arginine methyltransferase 5 (PRMT5), promoting hepatocellular carcinoma (HCC) cell proliferation, tumorigenicity, tumor invasion, and metastasis in vitro and in vivo [27]. As a member of the arginine methyltransferase (PRMTs) protein family, PRMT5 mediates the methylation of protein [28] and plays an important role of the progress in various cancer such as lung cancer, liver cancer, colorectal cancer, and breast cancer [29–32]. Our result also shows the LINC01128 highly expressed in the stage III-IV CRC, which may suggest that LINC01128 regulates PRMT5 function to aggravate the progress in stage III-IV CRC. However, the hypothesis must need more studies to confirm. ZEB1-AS1 (the antisense long noncoding RNA of zinc finger E-box-binding protein 1, ZEB1) can positively regulate the expression of ZEB1 that participates in cell apoptosis, chemoresistance, invasion, and metastasis in cancer [33–35], promoting the progress of HCC. In the correlation analysis, we also find that the three lncRNAs were associated with several m6A regulators, including RBM15, YDHTC2, FMR1, and FTO, which overexpress in the tumor tissues. That imply the three lncRNAs can exert their functions through m6A modification in CRC. Moreover, the three m6A-related lncRNAs also predict poor prognosis in cancer [27, 28, 33], which is consistent with our result that the patients with low-risk score have longer OS than the high-risk score. Therefore, we have sufficient reason to believe that the above three lncRNAs can serve as biomarkers for the prognosis of CRC.

Additionally, the prognosis of CRC is associated with immune cell like CD8+ T cell and dendritic cells (DCs) [36, 37]. Our result shows that the patients with low score get more DCs which is the potent antigen-presenting cells (APCs) that play a critical role in immunotherapy in CRC [38]. In functional analysis, we discover that the risk score is associated with cell adhesion molecule binding and cadherin binding. Different cell adhesion molecules express on the surface of DCs can present tumor antigens to T cells, which can indirectly facilitate T cell infiltration to kill the tumor cells [39]. Our results also showed that the ability to recruit DC was stronger in the low-risk group than in the high-risk group, but there was no significant difference in the ability to recruit CD8+ T cells between the two groups. That may indicate these lncRNAs possibly influence the prognosis through the regulation of DCs, suggesting that prognostic model with these three lncRNAs may be the potential biomarker for cancer. Nonetheless, the functions and interactions between tumor-immune cell and m6A-related lncRNAs need more investigating.

## 5. Conclusions

In summary, we proposed a novel prognostic model of m6A-related lncRNAs in CRC, which had an important prognostic value for the clinical evaluation of CRC patients. Future investigations on the mechanisms between these lncRNAs and m6A modification in CRC are needed.

## Data Availability

The data and materials used to support the findings of this study are available from the corresponding author upon request.
